# Systematic Pharmacology Reveals the Antioxidative Stress and Anti-Inflammatory Mechanisms of Resveratrol Intervention in Myocardial Ischemia-Reperfusion Injury

**DOI:** 10.1155/2021/5515396

**Published:** 2021-05-21

**Authors:** Zuzhong Xing, Qi He, Yinliang Xiong, Xiaomei Zeng

**Affiliations:** ^1^Brain Hospital of Hunan Province, The Second People's Hospital of Hunan Province, Changsha, Hunan, China; ^2^People's Hospital of Ningxiang City, Ningxiang, Hunan, China; ^3^Traditional Chinese Medicine Hospital of Ningxiang City, Ningxiang, Hunan, China

## Abstract

**Objective:**

To explore the oxidative stress and inflammatory mechanisms of resveratrol intervention in myocardial ischemia-reperfusion injury (MIRI).

**Methods:**

The potential targets of resveratrol were predicted by PharmMapper. The MIRI genes were collected by Online Mendelian Inheritance in Man (OMIM), GeneCards is used to collect related disease genes, and String is used for enrichment analysis. Animal experiments were then performed to verify the systematic pharmacological results. Hematoxylin-eosin (HE) staining was used to observe myocardial damage. The levels of serum interleukin-1*β* (IL-1*β*), IL-6, and tumor necrosis factor-*α* (TNF-*α*) in each experimental group were detected. The protein and mRNA expressions of Toll-like receptor 4 (TLR4), nuclear factor-kappa (NF-*κ*B) p65, IL-1*β*, IL-6, and TNF-*α* in rat myocardial tissue were measured.

**Results:**

The results of systematic pharmacology showed that insulin resistance, FoxO signaling pathway, adipocytokine signaling pathway, insulin signaling pathway, PI3K-Akt signaling pathway, ErbB signaling pathway, T-cell receptor signaling pathway, peroxisome proliferator-activated receptors (PPAR) signaling pathway, Ras signaling pathway, TNF signaling pathway, and so on were regulated to improve MIRI. The results of animal experiments showed that the myocardial cells of the sham operation group were arranged in fibrous form, and the myocardial ischemia-reperfusion injury group had obvious cell morphology disorder. Compared with the MIRI group, the resveratrol group had a certain degree of relief. Compared with the MIRI group, serum IL-1*β*, TNF-*α*, and IL-6 in the resveratrol group was significantly reduced (*P* < 0.05), and myocardial tissue TLR4, NF-*κ*B p65, IL-1*β*, IL-6, and TNF-*α* mRNA and protein expressions were significantly reduced (*P* < 0.05).

**Conclusion:**

Resveratrol can effectively improve MIRI, and its mechanism may be related to antioxidative stress and anti-inflammatory.

## 1. Introduction

Coronary artery disease (CAD) refers to heart disease caused by coronary artery atherosclerosis that causes lumen stenosis or occlusion, leading to myocardial ischemia, hypoxia, or necrosis, also known as ischemic heart disease [1]. When the larger branch of the coronary artery is completely occluded (or thrombosis), the myocardium supplied by this blood vessel becomes necrotic due to the lack of blood nutrition, and myocardial infarction (MI) will occur [2]. Myocardial infarction, as a high incidence of cardiovascular disease, is one of the diseases with the highest mortality rate in the world [3]. With the trend of population aging in the future, the prevalence and mortality of cardiovascular diseases will continue to rise for a long time, and the number of patients with cardiovascular diseases will continue to increase rapidly in the next 10 years [4].

The current effective strategy for the treatment of acute MI is to perform early coronary reperfusion through primary percutaneous coronary intervention (PCI) or thrombolysis [5, 6]. However, after the blood flow of the ischemic myocardium is restored, the myocardial injury does not alleviate and recover, but aggravates it, leading to other fatal injuries. This pathological syndrome is called myocardial ischemia-reperfusion injury (MIRI) [7, 8]. The factors leading to MIRI damage include inflammation, oxidative stress, calcium overload, mitochondrial permeability transition pore (mPTP) opening, and energy metabolism disorders [9–11]. The intervention of these pathological processes that lead to MIRI damage is a potential strategy to prevent cardiomyocyte death during MIRI [12, 13]. At present, natural plant ingredients have shown potential advantages in the treatment of MIRI [14]. Resveratrol, as a polyphenolic oxidant (nonflavonoid polyphenol organic compound), has been identified in the extracts of many plants and their fruits, such as grape (red wine), knotweed, peanut, and mulberry [15, 16]. The biological activity of resveratrol has antioxidative stress, anti-inflammatory, heart protection, immune regulation, antidiabetic, antiaging, and anticancer properties [17–19], especially playing an important role in the protection of myocardial damage [20, 21]. However, its molecular mechanism is not fully understood; especially, the biological network of resveratrol in regulating MIRI is still unknown. This study will first construct the biomolecular network of resveratrol intervention in MIRI through the strategy of systematic pharmacology and then further verify the molecular mechanism of resveratrol intervention in MIRI animal models.

## 2. Materials and Methods

### 2.1. Resveratrol Target Prediction and MIRI Disease Gene Acquisition

The structure of resveratrol was retrieved in PubChem (https://pubchem.ncbi.nlm.nih.gov/) and saved in sdf format. Resveratrol's sdf format file is imported into PharmMapper using reverse molecular docking technology to obtain predicted targets [22, 23]. GeneCards (http://www.genecards.org) [24] and OMIM database (http://omim.org/) [25] were utilized to obtain the MIRI genes [22]. Relevance score > 1 was used as the criterion to include MIRI-related genes when searching GeneCards. MIRI gene and resveratrol target protein were imported into UniProt (https://www.uniprot.org/) to get their official gene symbol (Table S1 and Table S2) [22].

### 2.2. Network Construction and Analysis Methods

The protein-protein interaction (PPI) data of resveratrol target and MIRI gene was collected from String (https://string-db.org/) [22, 26]. The String database was used to construct and analyze the PPI network. Database for Annotation, Visualization and Integrated Discovery (David) ver. 6.8 (https://david.ncifcrf.gov/) was used for Gene Ontology (GO) enrichment and Kyoto Encyclopedia of Genes and Genomes (KEGG) pathway enrichment analysis [22, 27]. String is used for Reactome pathway enrichment analysis.

### 2.3. Experimental Materials

#### 2.3.1. Experimental Animal

Ninety (90) clean-grade male Sprague Dawley (SD) rats were purchased from Hunan Slack Jingda Experimental Animal Co., Ltd., animal certificate number, Sheng Chan Xu Ke (SCX) (Xiang): 2017–0013). The rats weigh 290∼330 g, and they are kept in separate cages in a sterile animal breeding room after purchase. The temperature is controlled at 22∼26°C, the light and darkness are each 12 h, and the humidity is 40∼60%. All experiments were carried out in accordance with the “Guidelines for the Care and Use of Laboratory Animals” (National Institutes of Health Publication, No. 86–23, revised in 1996) and were approved by the Animal Ethics Committee of Hunan University of Chinese Medicine.

#### 2.3.2. Reagents and Instruments

Resveratrol (34092–100 MG) was purchased from Sigma Company in the United States. 3% sodium pentobarbital was obtained from China Pharmaceuticals Shanghai Chemical Reagent Company. Rat interleukin 1-beta (IL-1*β*) enzyme-linked immunoassay kit (ELISA) (bsk00027), tumor necrosis factor-*α* (TNF-*α*) kit (bsk00163), and IL-6 kit (bsk 0411) were purchased from Beijing Boaosen Biotechnology Co., Ltd. Creatine Kinase Isoenzyme-MB Isozyme (CK-MB) Assay Kit (E006-1-1), Lactate Dehydrogenase (LDH) Assay Kit (A020-2-2), Malondialdehyde (MDA) Assay Kit (A003-1-2), and Superoxide Dismutase (SOD) Assay Kit (A001-3-2) were purchased from Nanjing Jiancheng Institute of Biotechnology. Bicinchoninic acid (BCA) whole protein extraction kit (Cat. no.: 20170613) and BCA protein content detection kit (Cat. no.: 20170528) were purchased from Jiangsu Keygen Biotechnology Co., Ltd. Rabbit anti-Toll-like receptors (TLR)_4 polyclonal antibody (catalog number: ab13867), rabbit anti-*β*-actin antibody (catalog number: ab8227), and anti-histone H3 (methyl K37) antibody (catalog number: ab215728) were purchased from Abcam Inc. Rabbit anti-nuclear factor-kappa (NF-*κ*B) p65 monoclonal antibody was purchased from Cell Signaling Technology. Horseradish enzyme-labeled secondary antibody was purchased from Beijing Zhongshanjinqiao Biotechnology Company (catalog number: ZB2306). Polyvinylidene fluoride (PVDF) membrane (0.45 *μ*m, product number: 101123-1) was purchased from Shanghai Yantuo Biotechnology Co., Ltd.; primers were obtained from Shanghai Biotech; RNA extraction kit was purchased from Tiangen Biochemical Technology (Beijing) Co., Ltd., (DP419). TransScript One-Step gDNA Removal and cDNA Synthesis SuperMix Kit (Cat. no.: J21201), 2 x EcoTaq PCR SuperMix Kit (Cat. no.: L20719), TransStart Tip Green qPCR SuperMix Kit (Cat. no.: K31213), and Gelstain staining solution (Cat. no.: GS101) were purchased from TransGen Biotech Inc.

HX-300 animal ventilator and BL-420 biological signal acquisition system were purchased from Chengdu Taimeng Technology Co., Ltd. Visible fluorescence imager was purchased from Azure Biosystems, USA; SDS-PAGE electrophoresis instrument, Quantity One 4.6.2 image analyzer, and electromembrane transfer instrument were obtained from Bio-Rad Inc.; SW-CJ2-1F ultraclean table was purchased from Suzhou Antai Bioscience and Technology Company; and EASYCYCLER 96 PCR instrument was obtained from Jena Company.

### 2.4. Experimental Methods

#### 2.4.1. Animal Modeling Methods

The rats were fasted for 12 hours before the operation, and the rats were anesthetized by intraperitoneal injection of 3% pentobarbital sodium 30 mg/kg. The rat was fixed on the operating table in the supine position and connected to the BL-420 biological function system, electrocardiograph (ECG) standard II lead, tracheal intubation, and ventilator-assisted breathing were recorded, the third and fourth ribs on the left side of the sternum were incised, and the pericardium was separated. Then the left anterior descending coronary artery (LAD) was ligated with 6.0 silk thread, and both ends of the thread were threaded into the PE tube, and the polyethylene (PE) tube was clamped with a needle holder and pushed forward to cause cardiac compression ischemia. If ECG monitoring shows that the ST segment is significantly elevated, it is considered to be successful. After 30 minutes of ischemia, the PE tube was released and the PE tube was reperfused for 3 hours. The rat's ECG showed significant elevation of the ST segment, that is, the MIRI model was successfully constructed, and then blood was taken from the abdominal aorta; the left ventricular tissue of the heart was taken.

#### 2.4.2. Animal Grouping and Intervention

First, resveratrol was dissolved in dimethyl sulfoxide and diluted with saline. After 1 week of adaptive feeding, the rats were divided into five groups according to the random number method, with 18 rats in each group: sham group: the LAD coronary artery of the heart is only threaded and not ligated. MIRI group: myocardial ischemia is 45 minutes and reperfusion is 3 hours. Low-resveratrol, high-dose preconditioning + MIRI group: resveratrol 40 mg/kg or 80 mg/kg was injected intraperitoneally before myocardial ischemia-reperfusion injury. The sham operation group and MIRI group were pretreated with the same dose of normal saline for intraperitoneal injection. Rats of different groups were raised in separate cages, and each rat was labeled with picric acid serial number on the limbs.

#### 2.4.3. Echocardiography

Before and 3 hours after making the model, the rats were intraperitoneally anesthetized with 3% sodium pentobarbital (30 mg/kg), and the two-dimensional ultrasound system was used to detect the cardiac function of the rats. A probe with a frequency of 10 MHz and a depth of 2 cm is placed on the animal's left chest. The short-axis papillary muscles are recorded on 2D ultrasound, and the left ventricle and tissue Doppler imaging is recorded from the level of the papillary muscles with M-mode ultrasound. Left ventricular ejection fraction (LVEF) and left ventricular fractional shortening (LVFS) were measured.

#### 2.4.4. Animal Specimen Collection and Processing

After the completion of the perfusion, blood was drawn from the abdominal aortic artery. After collection, the blood was centrifuged in a low-temperature high-speed centrifuge at 4°C and 3000 g for 15 minutes, and the serum was separated. After the serum was collected, it was placed in a −20°C low-temperature refrigerator for the detection of biochemical indicators. After blood collection, the rats were sacrificed by neck dislocation, and the myocardial tissue was quickly taken out, washed with normal saline, partly fixed with 4% paraformaldehyde, and partly stored in an ultralow-temperature refrigerator (−80°C) for testing.

#### 2.4.5. Pathological Observation

The heart was uniformly cut about a quarter of the apex and placed in 4% neutral formalin solution for fixation. After 48 hours, it was taken out for dehydration and paraffin-embedded section. Then, the slices were soaked in xylene I and xylene II for 5 minutes, soaked in absolute ethanol I and absolute ethanol II for 2 minutes each, and soaked in 95% ethanol, 85% ethanol, and 75% ethanol for 3 minutes. After the sections were immersed in distilled water, they were stained with hematoxylin for 5 min, differentiated with 1% hydrochloric acid for 30 s, soaked in warm water for 10 min and eosin for 2 min, washed with three distilled water, dehydrated, transparent, mounted with neutral gum, and dried overnight. The sections were observed under an optical microscope and filmed.

#### 2.4.6. Detection of Serum LDH and CK-MB

The inflammatory factors IL-1*β*, IL-6, and TNF-*α* are determined by ELISA double-antibody sandwich method. All operations are carried out in strict accordance with the instructions provided by the kit. The blood collected from the abdominal aorta was centrifuged at 3000 r/min for 10 min, and the serum was collected and stored at −20°C. The detection steps were performed in strict accordance with the ELISA kit instructions, using double-well determination, measuring the absorbance (OD) value of each well, and calculating the concentration of IL-1*β*, IL-6, and TNF-*α* according to the standard curve.

#### 2.4.7. Detection of Myocardial Tissue Glutathione Peroxidase (GSH-Px), MDA, and SOD

The myocardial tissue was ground into a homogenate with a glass homogenizer, and then the protein concentration of each group of cells was measured according to the instructions of the BCA kit. The working fluid is then configured according to the kit instructions. Then, the reaction initiation solution in the kit is melted and mixed, and diluted according to the ratio of the reaction initiation solution of each kit to the detection buffer of each sample solution, and the mixed liquid is the reaction initiation working solution. Then, the sample measurement is performed: the sample wells and control wells are set up in a 96-well plate, the sample and working solution are added, and finally, the reaction starter solution is added. The sample was incubated at 37°C for 30 minutes. Finally, the absorbance at 532 nm and 560 nm was measured with a microplate reader, and the GSH-Px, SOD, and MDA values were calculated.

#### 2.4.8. Detection of TLR4, NF-*κ*B p65, IL-1*β*, TNF-*α*, and IL-6 mRNA Expression in Myocardial Tissue

50 mg of the infarct area of the heart tissue was selected, and the total RNA was extracted with the total RNA kit. After extraction, it was quantified and leveled, and then each group took 20 *μ*g for reverse transcription. After the reverse transcription is completed, the amplification reaction can be carried out. The amplification conditions are as follows: 95°C predenaturation for 2 min; 95°C denaturation for 10 s, 56°C annealing for 30 s, 72°C extension for 35 s, repeated cycles of 38 times, and finally 72°C extension for 5 min. The results were calculated according to (2 − ΔΔCt): ΔCt value = Ct value target gene − Ct value GAPDH; ΔΔCt = ΔCt value of experimental group − ΔCt value of the sham group; ratio = 2 − ΔΔCt. The primer is shown in Table 1.

#### 2.4.9. Detection of TLR4, NF-*κ*B p65, IL-1*β*, TNF-*α*, and IL-6 Protein Expression in Myocardial Tissue

500 mg of myocardial tissue was extracted with a tissue total protein extraction kit. After completion, it was quantified and leveled. After that, 60 *μ*g of each group was heated and denatured. Then SDS-PAGE was performed. After the electrophoresis is completed, the required strips are cut and transferred to the PVDF membrane, and the PVDF membrane is sealed with skim milk for 2 hours. After washing three times, the PVDF membrane was placed in the primary antibody diluent (TLR4, NF-*κ*B p65, beta-actin, and histone H3 were diluted at a ratio of 1 : 500, and IL-1*β*, IL-6, and TNF-*α* were all 1 : 200), shaken at room temperature for 1 h, and put in 4°C environment overnight. After the primary antibody was recovered, the PVDF membrane was washed 4 times with Tris-buffered saline with Tween (TBST) (1 time in 15 minutes and 3 times in 5 minutes). The PVDF membrane was then placed in the secondary antibody diluent on a horizontal shaker at room temperature for 2 h, and the membrane was washed 4 times. Finally, the PVDF film was completely immersed in the luminescent liquid for about 30 s (protected from light), and then the film was placed in a UVP gel imaging exposure instrument for development. ImageJ software is used to analyze the ratio of the gray value of the target protein band to the *β*-actin band, which reflects the protein expression level.

### 2.5. Statistical Analysis

Statistical analysis was performed using SPSS 20.0 software package, GraphPad prism software 7.0 was used for graphing, and the measured results were expressed by mean + SEM. The data of multiple groups were compared by one-way analysis of variance, and the pairwise comparison between groups was mostly by the SKN-q method, and *P* < 0.05 was considered statistically significant.

## 3. Results

### 3.1. Resveratrol-MIRI PPI Network Analysis

#### 3.1.1. Resveratrol-MIRI PPI Network Construction

**Figure 1:**
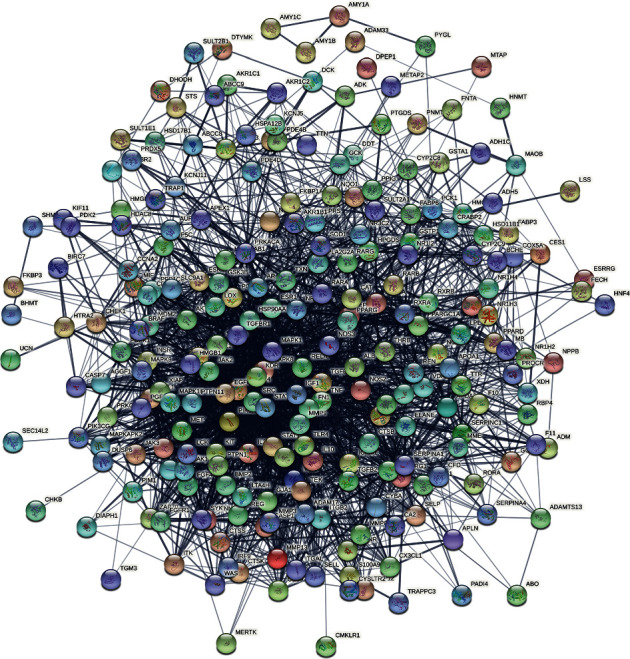
Resveratrol-MIRI PPI Network.

A total of 171 resveratrol targets and 93 MIRI genes were obtained. They were input into String to construct Resveratrol-MIRI PPI Network (Figure 1). In this network, the top 30 nodes in degree are as follows: ALB (127 edges), TNF (110 edges), MAPK1 (99 edges), EGFR (96 edges), SRC (95 edges), FN1 (93 edges), STAT3 (92 edges), MAPK8 (84 edges), IGF1 (84 edges), MMP9 (81 edges), ESR1 (77 edges), HSP90AA1 (76 edges), IL10 (72 edges), NOS3 (72 edges), MAPK14 (72 edges), TLR4 (71 edges), CAT (70 edges), ICAM1 (60 edges), IL2 (58 edges), RELA (57 edges), STAT1 (57 edges), KDR (57 edges), PPARG (55 edges), MMP2 (54 edges), MPO (53 edges), PIK3R1 (53 edges), JAK2 (50 edges), AR (50 edges), IFNG (48 edges), and NR3C1 (48 edges).

#### 3.1.2. Enrichment Analysis of Resveratrol-MIRI PPI Network

**Figure 2:**
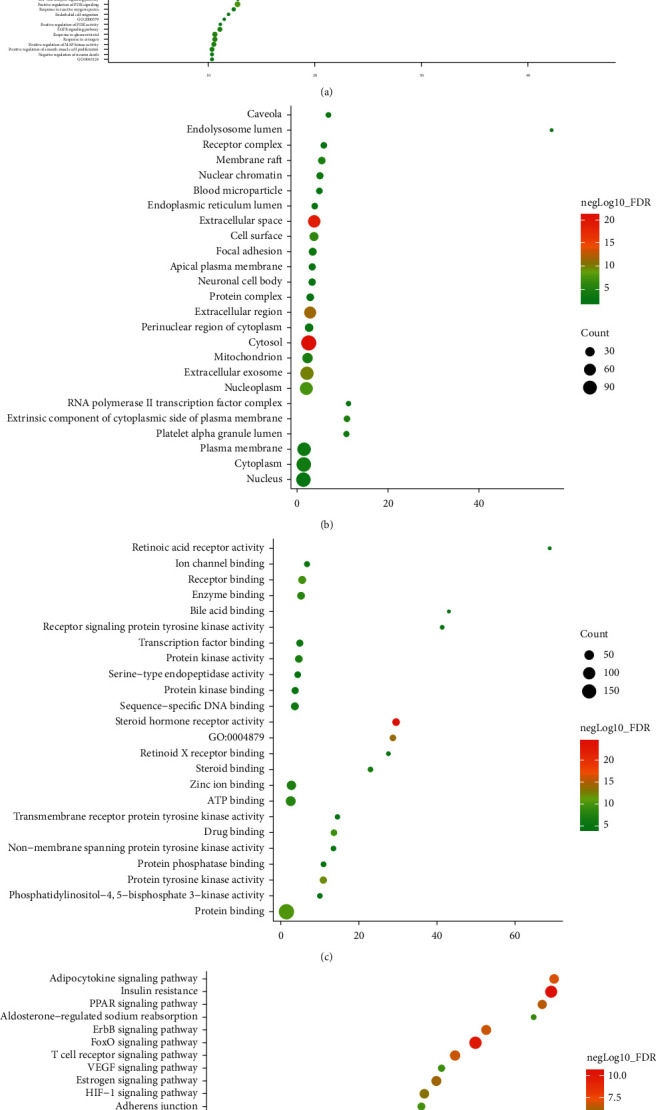
Bubble chart of enrichment analysis. (a) Biological processes; (b) cell components; (c) molecular function; (d) signaling pathways; *X*-axis is fold enrichment.

**Figure 3:**
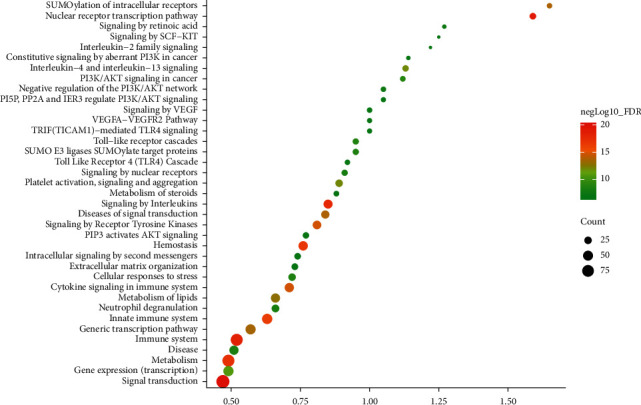
Bubble chart of Reactome pathway (*X*-axis is strength).

**Figure 4:**
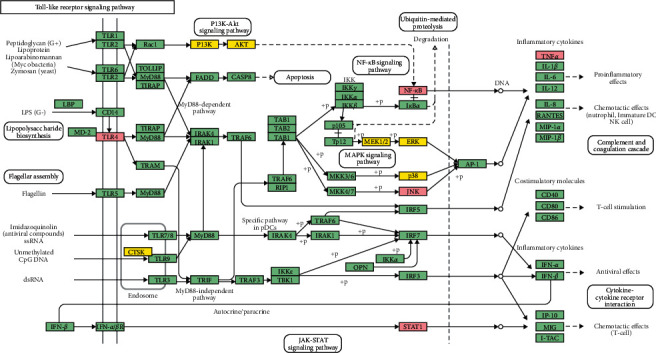
Toll-like receptor signaling pathway adapted from KEGG (ID: hsa04620) (MIRI gene is marked in pink, and the resveratrol target is marked in yellow).

The targets and genes in Resveratrol-MIRI PPI Network are imported into DAVID and String for enrichment analysis. A total of 89 MIRI-related biological processes, 25 MIRI-related cell components, 24 MIRI-related molecular functions, 31 MIRI-related signaling pathways, and 37 MIRI-related Reactome pathways were returned (Table S3 and Table S4 and Figure 2). The biological processes include steroid hormone-mediated signaling pathway, cellular response to lipopolysaccharide, negative regulation of the apoptotic process, positive regulation of ERK1 and ERK2 cascade, positive regulation of cell proliferation, aging, leukocyte migration, peptidyl-tyrosine phosphorylation, intracellular receptor signaling pathway, positive regulation of gene expression, positive regulation of nitric oxide (NO) biosynthetic process, positive regulation of PI3K signaling, response to hypoxia, activation of mitogen-activated protein kinase (MAPK) activity, positive regulation of vasodilation, and so on. The cell components include cytosol, extracellular space, extracellular region, extracellular exosome, nucleoplasm, cell surface, extrinsic component of cytoplasmic side of plasma membrane, membrane raft, mitochondrion, cytoplasm, platelet alpha granule lumen, nuclear chromatin, plasma membrane, focal adhesion, and so on. The molecular functions include steroid hormone receptor activity, protein tyrosine kinase activity, protein binding, receptor binding, drug binding, enzyme binding, ATP binding, zinc ion binding, protein kinase activity, steroid binding, and so on. The signaling pathways include insulin resistance, FoxO signaling pathway, adipocytokine signaling pathway, insulin signaling pathway, PI3K-Akt signaling pathway, ErbB signaling pathway, T-cell receptor signaling pathway, PPAR signaling pathway, Ras signaling pathway, estrogen signaling pathway, TNF signaling pathway, and so on. The Reactome pathways include signal transduction, nuclear receptor transcription pathway, immune system, signaling by interleukins, metabolism, hemostasis, innate immune system, cytokine signaling in the immune system, signaling by receptor tyrosine kinases, diseases of signal transduction, SUMOylation of intracellular receptors, generic transcription pathway, metabolism of lipids, and so on. The fold enrichment, count, and false discovery rate (FDR) of the biological processes, cell components, molecular functions, and signaling pathways are shown in Figure 2. The strength, FDR, and count of the Reactome pathway are shown in Figure 3. The targets and genes in Toll-like receptor signaling pathway are shown in Figure 4 as an example.

### 3.2. Effects of Resveratrol on Heart Function

**Figure 5:**
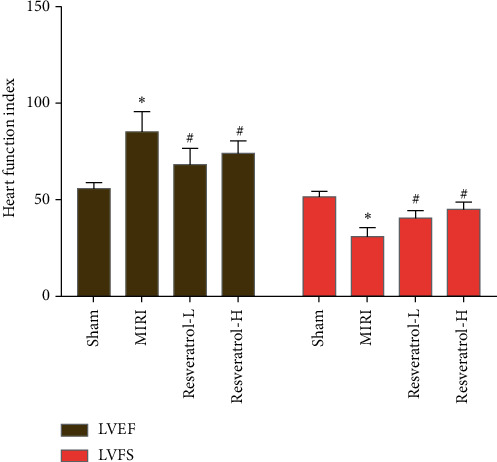
Effects of resveratrol on heart function (*∗*compared with the sham operation group, *P* < 0.05; ^#^compared with the MIRI group, *P* < 0.05).

Before operation and 24 hours after operation, echocardiography showed that the heart function of rats in each group did not change significantly before operation, and there was no statistical difference. Echocardiogram at 24 hours after operation showed that LVFS and LVEF were lower in the sham group than in the MIRI group. LVFS and LVEF increased significantly after treatment with resveratrol (*P* < 0.05). The above results indicate that resveratrol improves heart dysfunction caused by MIRI (Figure 5).

### 3.3. Histopathological Changes

**Figure 6:**
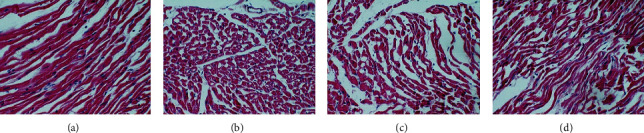
Histopathological changes (HE staining 100X). (a) Sham. (b) MIRI. (c) Res-L. (d) Res-H.

In the sham operation group, the morphology of the cells was tightly arranged in a fibrous shape without inflammatory factors. In the MIRI group, there was an obvious disorder of cell morphology, the horizontal stripes disappeared, and there was some inflammatory cell infiltration. Compared with the MIRI group, the other groups have a certain degree of relief; especially, the resveratrol high-dose group has the most obvious improvement, but there is still a certain difference compared with the sham operation group (Figure 6).

### 3.4. Effects of Resveratrol on Myocardial Injury Markers

**Figure 7:**
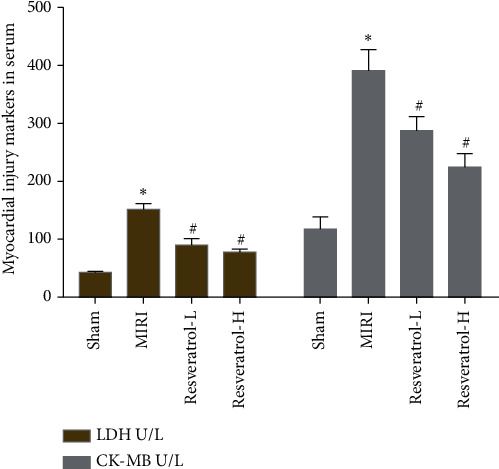
Effects of resveratrol on myocardial injury markers (*∗*compared with the sham operation group, *P* < 0.05; ^#^compared with the MIRI group, *P* < 0.05).

The CK-MB and LDH values in the sham group were lower, and the CK-MB and LDH values in the MIRI group were significantly increased. Compared with the model group, resveratrol was significantly reversed after pretreatment, and the resveratrol high-dose group was significantly lower than the low-dose group (Figure 7).

### 3.5. Effects of Resveratrol on Myocardial Tissue MDA, GSH-Px, and SOD

**Figure 8:**
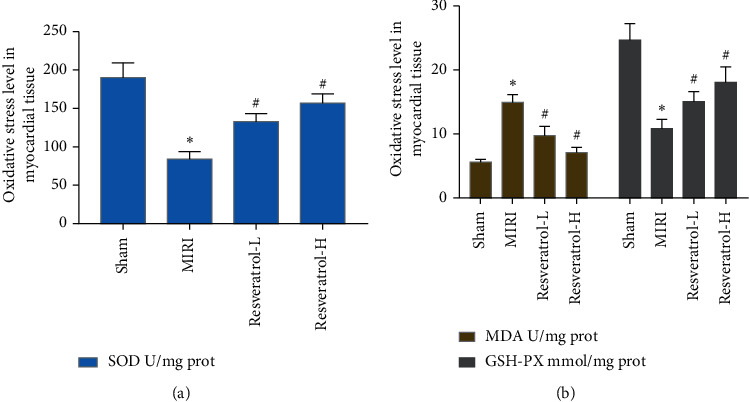
Effects of resveratrol on myocardial tissue MDA, GSH-Px, and SOD (*∗*compared with the sham operation group, *P* < 0.05; ^#^compared with the MIRI group, *P* < 0.05).

The level of MDA in the myocardial tissue of rats in the MIRI group increased significantly (*P* < 0.05), and the activities of GSH-Px and SOD decreased significantly (*P* < 0.05). The MDA level of the resveratrol group was significantly lower than that of the MIRI group (*P* < 0.05), and the activities of GSH-Px and SOD were significantly increased (*P* < 0.05) (Figure 8).

### 3.6. Effects of Resveratrol on Serum IL-1*β*, TNF-*α*, and IL-6

**Figure 9:**
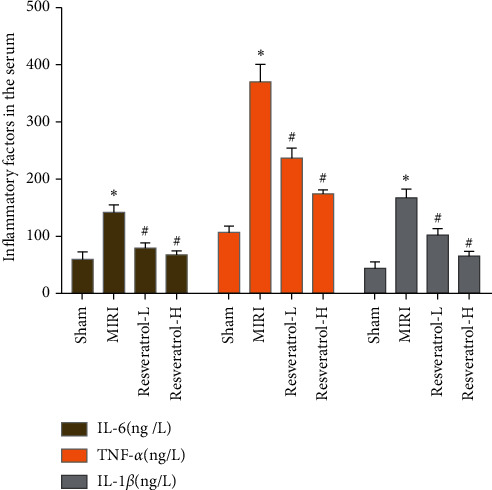
Effects of resveratrol on serum IL-1*β*, TNF-*α*, and IL-6 (*∗*compared with the sham operation group, *P* < 0.05; ^#^compared with the MIRI group, *P* < 0.05).

Compared with the sham group, the levels of IL-1*β*, TNF-*α*, and IL-6 in the MIRI group increased significantly (*P* < 0.05). Compared with the MIRI group, the levels of IL-1*β*, TNF-*α*, and IL-6 in the high and low doses of resveratrol decreased significantly (*P* < 0.05). Among them, compared with the low-dose group, the high-dose group was also significantly reduced (*P* < 0.05), indicating that resveratrol has the anti-inflammatory ability (Figure 9).

### 3.7. Effects of Resveratrol on the Expression of Myocardial Tissue TLR4, NF-*κ*B p65, IL-1*β*, TNF-*α*, and IL-6 mRNA and Protein

#### 3.7.1. Effects of Resveratrol on Myocardial Tissue TLR4 and NF-*κ*B p65 mRNA

**Figure 10:**
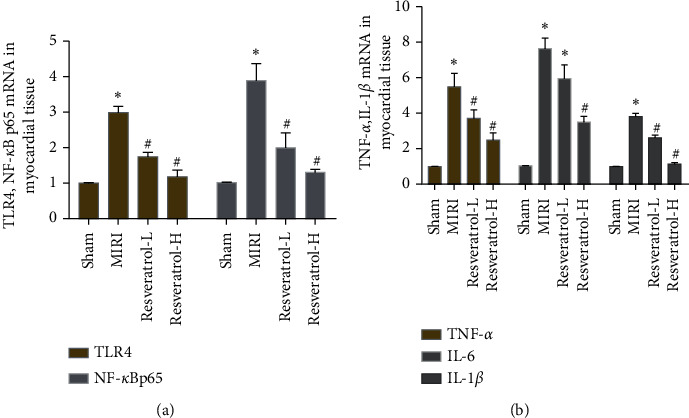
Effects of resveratrol on the expression of myocardial tissue TLR4, NF-*κ*B p65, IL-1*β*, TNF-*α*, and IL-6 mRNA (*∗*compared with the sham operation group, *P* < 0.05; ^#^compared with the MIRI group, *P* < 0.05).

Compared with the sham operation group, the expression of TLR4 and NF-*κ*B P65 mRNA in the MIRI group increased (*P* < 0.05). Compared with the MIRI group, the expression of TLR4 and NF-*κ*B P65 mRNA in the resveratrol low-dose and high-dose groups was significantly reduced (*P* < 0.05). In addition, the TLR4 and NF-*κ*B P65 mRNA in the resveratrol high-dose group were lower than those in the resveratrol low-dose group (*P* < 0.05) (Figure 10).

#### 3.7.2. Effects of Resveratrol on Myocardial Tissue IL-1*β*, TNF-*α*, and IL-6 mRNA

Compared with the sham group, the expression levels of IL-1*β*, TNF-*α*, and IL-6 mRNA in the MIRI group increased (*P* < 0.05). Compared with the MIRI group, the levels of IL-1*β*, TNF-*α*, and IL-6 mRNA in the resveratrol group were decreased (*P* < 0.05) (Figure 10).

#### 3.7.3. Effects of Resveratrol on Myocardial Tissue TLR4 and NF-*κ*B p65 Protein

**Figure 11:**
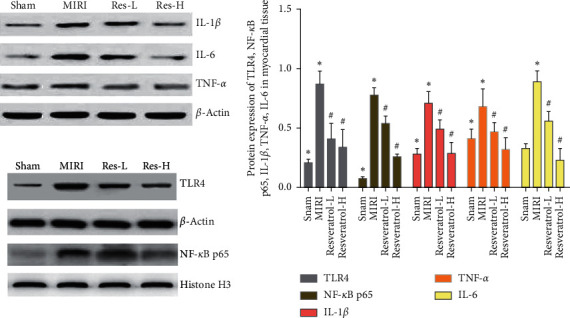
Effects of resveratrol on the expression of myocardial tissue TLR4, NF-*κ*B p65, IL-1*β*, TNF-*α*, and IL-6 protein (*∗*compared with the sham operation group, *P* < 0.05; ^#^compared with the MIRI group, *P* < 0.05).

Compared with the sham operation group, the expression of TLR4 and NF-*κ*B P65 protein in the MIRI group increased (*P* < 0.05). Compared with the MIRI group, the expression of TLR4 and NF-*κ*B P65 protein in the resveratrol low-dose and high-dose groups was significantly reduced (*P* < 0.05) (Figure 11).

#### 3.7.4. Effects of Resveratrol on Myocardial Tissue IL-1*β*, TNF-*α*, and IL-6 Protein

Compared with the sham group, the expression levels of IL-1*β*, TNF-*α*, and IL-6 protein in the MIRI group increased (*P* < 0.05). Compared with the MIRI group, the levels of IL-1*β*, TNF-*α*, and IL-6 protein in the resveratrol group were decreased (*P* < 0.05) (Figure 11).

## 4. Discussion

With the rapid development of medical technology, the continuous popularization and promotion of PCI, coronary artery bypass grafting (CABG), and coronary thrombolysis technologies and drugs have brought great help to patients with ischemic cardiomyopathy [28]. But in clinical, MIRI is an unavoidable problem, which is also a problem that needs to be solved urgently in clinical practice. Reperfusion injury is the key to determining myocardial viability after ischemic myocardial blood flow reconstruction. Existing studies have found that ischemia-reperfusion can exacerbate the irreversible cell necrosis of damaged cardiomyocytes [29, 30]. Studies of cellular molecular mechanisms have shown that there is a causal relationship between MIRI and intracellular calcium overload, inflammation, and oxidative stress [13, 31, 32]. Mitochondrial dysfunction is considered to be the main source of reactive oxygen species in the pathogenesis of reperfusion injury, and it also promotes inflammation and endothelial damage [33]. At the same time, the opening of mPTP and subsequent release of cytochrome c from damaged mitochondria triggers the intrinsic apoptotic process by activating the caspase-9/3 signaling pathway, leading to cardiomyocyte apoptosis. Apoptosis combined with other programmed cell deaths together leads to the expansion of the infarct size after MIRI injury [34]. In addition to the apoptotic pathway, autophagy, a process of programmed cell death caused by energy metabolism after ischemia, plays an important role in the occurrence of reperfusion injury. The activation or inhibition of autophagy may have beneficial or harmful effects in the case of MIRI [35]. In addition, platelet aggregation caused by MIRI leads to microvascular obstruction, which is characterized by microcirculation spasm, intraluminal thrombosis, and obvious endothelial cell swelling and dysfunction, which ultimately leads to slow blood flow [36]. After reperfusion injury, inflammation and oxidative stress mainly lead to a large loss of cardiomyocytes, which leads to the expansion of the infarct area. Among them, neutrophils are also closely related to the inflammatory response, and the large accumulation of neutrophils in the reperfusion area will cause a negative impact on the survival of cardiomyocytes [37]. In addition, damage to mitochondria and immune cell infiltration can lead to a large amount of ROS generation, which leads to excessive oxidative stress [38].

In this study, we found that resveratrol can regulate steroid hormone-mediated signaling pathway, cellular response to lipopolysaccharide, negative regulation of the apoptotic process, positive regulation of ERK1 and ERK2 cascade, positive regulation of cell proliferation, aging, leukocyte migration, peptidyl-tyrosine phosphorylation, intracellular receptor signaling pathway, insulin resistance, FoxO signaling pathway, adipocytokine signaling pathway, insulin signaling pathway, PI3K-Akt signaling pathway, ErbB signaling pathway, T-cell receptor signaling pathway, PPAR signaling pathway, Ras signaling pathway, estrogen signaling pathway, TNF signaling pathway, and so on. Previous studies also provided many evidences that resveratrol protects MIRI. For example, in terms of inflammation, it shows that resveratrol protects the myocardium by inactivating NALP3 inflammasomes and inhibiting the inflammatory cascade mediated by IL-1*β* and IL-18 [39]. Evidence from previous studies shows that resveratrol is an antioxidant that can regulate the multistep process of redox stress [40]. In vivo and in vitro MIRI models, resveratrol pretreatment reduces ROS levels, inhibits the formation of MDA, and is negatively correlated with the increase in antioxidant enzyme expression [41, 42]. Meanwhile, inflammation and/or oxidative stress trigger the apoptotic cascade through internal or external apoptotic signaling pathways, leading to cardiomyocyte apoptosis and poor ventricular remodeling and further worsening the contractile function after MIRI [43]. Existing studies have also shown that resveratrol significantly reduces apoptosis in MIRI heart models by eliminating ROS production and inhibiting inflammation [44]. Resveratrol can also prevent the opening of mPTP, the release of cytochrome c from the mitochondria, and subsequent activation of caspase-3 during I/R injury, thereby preventing cell death caused by mitochondrial dysfunction [20]. In terms of angiogenesis caused by myocardial ischemia-reperfusion, resveratrol reduces MIRI by upregulating vascular endothelial growth factor B [42]. In terms of endothelial dysfunction caused by MIRI, resveratrol inhibits I/R-induced iNOS and upregulates the expression of eNOS and nNOS to improve myocardial ischemia-reperfusion injury [45]. When NOS inhibitors (NG-nitro-L-arginine methyl ester, L-NAME) or cGMP inhibitors (MB) are used, the cardioprotective effects of resveratrol are counteracted. This indicates that resveratrol is essential in promoting angiogenesis and inhibiting vascular endothelial dysfunction [46]. In terms of oxidative stress, resveratrol can reduce myocardial oxidative stress by stimulating Sirtuin1 (SIRT1) or inhibiting GSK3*β* in diabetic cardiac ischemia-reperfusion injury models and increasing nuclear factor E2-related factor 2 (Nrf2) expression [47]. In terms of apoptosis, resveratrol protects myocardial cell apoptosis from ischemia-reperfusion injury by regulating the phosphorylation level of proteins relative to the PI3K/Akt/e-NOS pathway [48]. In terms of autophagy, resveratrol can reduce the ischemia/reperfusion injury of diabetic myocardium by inducing autophagy [49]. In terms of calcium signal pathway, resveratrol inhibits STIM1-induced intracellular Ca^2+^ accumulation, exerts antiapoptotic activity, and improves cardiac function recovery after MIRI [50].

The mechanism of ischemia-reperfusion injury and therapeutic drugs has positive significance for the treatment of patients with heart disease for noncardiac surgery. Current studies have shown that the main signaling pathways of MIRI are Ca^2+^ signaling pathway, mTOR signaling pathway, JAK-STAT signaling pathway, MAPK signaling pathway, NRF2 signaling pathway, JAK-STAT signaling pathway, SIRT signaling pathway, nitric oxide signaling pathway, TLR4/NF-kB signaling pathway, etc. [51–55]. Current studies have shown that the rapid increase of TLR4 and NF-kB can occur during transient cardiac ischemia. In addition, the two did not decrease after reperfusion, but increased further [56]. It was found in animal models of acute MIRI that the TLR4/NF-kB signaling pathway is closely related to cardiomyocyte apoptosis. Reactivation of the TLR4/NF-kB signaling pathway can directly induce the occurrence of cardiomyocyte apoptosis, and inhibiting the expression of TLR4 and NF-xB can inhibit apoptosis [57]. Further studies have shown that the activation of TLR4 and NF-kB in cardiomyocytes can lead to a significant increase in downstream inflammatory factors TNF-*α* and IL-6, which in turn directly cause damage to myocardium and vascular endothelial cells. Similarly, resveratrol inhibits the inflammatory response after MIRI by inhibiting the expression of TLR4 and NF-kB, thereby reducing myocardial ischemia-reperfusion injury [58]. Our research also observes the changing characteristics of the above indicators. Compared with the sham operation group, the myocardial ischemia-reperfusion injury group showed significant increases in TLR4, NF-KB, IL-1*β*, TNF-*α*, and IL-6. Resveratrol can effectively reduce the expression of the above five indicators. In addition, resveratrol can also regulate oxidative stress markers (such as MDA, GSH, and SOD) in myocardial tissue. Therefore, resveratrol may inhibit the expression of TLR4 and NF-kB in the myocardium, thereby inhibiting the production of inflammatory factors IL-1*β*, TNF-*α*, and IL-6 in the myocardium, and ultimately protect myocardial cells.

## 5. Conclusion

Resveratrol can effectively improve MIRI, and its mechanism of action may be related to reducing the expression of TLR4/NF-kB in the myocardium, thereby improving inflammation. However, this study also has certain limitations, that is, MIRI is the result of multiple effects, involving multiple signal pathways. In the future, we will conduct further in-depth research on other signal pathways.

## Figures and Tables

**Table 1:** The primer.

Gene	Direction	Sequence
TLR4	Forward primer	5′-GCCTTTCAGGGAATTAAGCTCC-3′
	Reverse primer	5′-GATCAACCGATGGACGTGTAAA-3′

NF-*κ*B p65	Forward primer	5′-ATGGCAGACGATGATCCCTAC-3′
	Reverse primer	5′-CGGAATCGAAATCCCCTCTGTT-3′

IL-1*β*	Forward primer	5′-TTGGGCTGTCCAGATGAGAG-3′
	Reverse primer	5′-CACACTAGCAGGTCGTCATCAT-3′

TNF-*α*	Forward primer	5′-GTGCCTCAGCCTCTTCTCATT-3′
	Reverse primer	5′-CCAGTTGGTTGTCTTTGAGATCC-3′

IL-6	Forward primer	5′-GTATGAACAGCGATGATGCACT-3′
	Reverse primer	5′-AACTCCAGAAGACCAGAGCAG-3′

*β*-Actin	Forward primer	5′-GACTATGACTTGAATGCGGTCC-3′
	Reverse primer	5′-TCAGCACCCAAAGTCACCAAGT-3′

## Data Availability

The data that support the findings of this study are openly available in supplementary materials.

## Supplementary Materials

Supplementary Materials Table S1: predicted potential targets of resveratrol. Table S2: MIRI genes. Table S3: enrichment analysis of Resveratrol-MIRI PPI Network. Table S4: Reactome pathway of Resveratrol-MIRI PPI.
