# Supplementary material for: Systematic Pharmacology Reveals the Antioxidative Stress and Anti-Inflammatory Mechanisms of Resveratrol Intervention in Myocardial Ischemia-Reperfusion Injury
Source: Evid Based Complement Alternat Med. 2021 May 21;2021:5515396. doi: 10.1155/2021/5515396 (PMC8163539; doi:10.1155/2021/5515396)
Supplement: Supplementary Materials — Table S1: predicted potential targets of resveratrol. Table S2: MIRI genes. Table S3: enrichment analysis of Resveratrol-MIRI PPI Network. Table S4: Reactome pathway of Resveratrol-MIRI PPI. [file 5515396.f1.zip › 5515396.f1/Table S1.pdf]

**Table S1 Predicted potential targets of Resveratrol**

BMP2
STS
ALB
AKR1C2
APOA2
KIF11
PDPK1
MMP3
PIK3CG
NR3C2
MAPK14
PPP5C
ADAM17
ESR1
GC
MAPK10
MAPK1
CA2
FNTA
F2
BCHE
SRC
CES1
DPP4
PIM1
PDE4B
DUSP6
CTNNA1
MMP8
METAP2
AKR1B1
FGFR1
CFD
BACE1
PDE4D
AR
MAOB
SULT2A1
PGF
HSD11B1
PTPN11
RORA
AKR1C1
TTPA
AKR1C3
HSD17B1
TTR

PPARD
NQO1
SEC14L2
WAS
GSK3B
PCK1
ADH5
CDK2
MMP13
PRKACA
ESR2
EGFR
ADH1C
CHEK1
GSTP1
GSTA1
MDM2
PPARA
SHBG
THRB
ADK
KDR
PTPN1
TGFBR1
TRAPPC3
NR1H3
ELANE
PGR
CYP2C9
SHMT1
HPGDS
NR1H2
PDK2
MET
LCK
DHODH
DCK
CCNA2
JAK3
F10
PRKCQ
ZAP70
MTAP
RXRA
MMP2
HDAC8
SYK
FKBP1A

RBP4
FABP3
FABP6
ITK
HNF4G
SERPINA1
ESRRG
FABP7
PYGL
REN
SULT2B1
NR3C1
LSS
NR1I2
PADI4
SULT1E1
GCK
HSP90AA1
AKT2
CTSS
AURKA
MMP12
ERBB4
ITGAL
S100A9
PLA2G2A
PPARG
DPEP1
NR1H4
XIAP
RARG
MAPKAPK2
HMGCR
TGM3
F11
CRABP2
CYP2C8
FECH
RARA
BRAF
IGF1
MME
HNMT
IL2
TEK
MAP2K1
ABL1
THRA

VDR
KIT
RARB
CTSB
CTSK
LTA4H
ARG1
HSP90AB1
PIK3R1
RXRB
TGFB2
ABO
MMP9
ADAM33
BHMT
INSR
PPP1CC
CASP1
BIRC7
TPSB2
PCTP
DTYMK
PROCR
PNMT
FKBP3
AMY1A
AMY1B
AMY1C
