# Supplementary material for: Systematic Pharmacology Reveals the Antioxidative Stress and Anti-Inflammatory Mechanisms of Resveratrol Intervention in Myocardial Ischemia-Reperfusion Injury
Source: Evid Based Complement Alternat Med. 2021 May 21;2021:5515396. doi: 10.1155/2021/5515396 (PMC8163539; doi:10.1155/2021/5515396)
Supplement: Supplementary Materials — Table S1: predicted potential targets of resveratrol. Table S2: MIRI genes. Table S3: enrichment analysis of Resveratrol-MIRI PPI Network. Table S4: Reactome pathway of Resveratrol-MIRI PPI. [file 5515396.f1.zip › 5515396.f1/Table S2.pdf]

Table S2 MIRI Gene

NOL3
SOD1
TNF
ICAM1
KCNJ5
MAPK8
SELP
GJA1
MB
MALAT1
NOS3
NOS2
TXN
ADM
JAK2
CAT
EPRS
KNG1
MMP2
AKR1B1
ADAMTS13
NPPB
SLC9A3R2
MPO
MERTK
APOA1
MIF
KCNJ11
XDH
CHKB
HMGB1
APLNR
SELL
UCN
APLN
PPARG
STAT1
STAT3
TLR4
JAK1
CASP7
FN1
NOS1
RELA
IFNG
EPAS1
DIAPH1

TTN
XIAP
IL10
HTRA2
ABCC8
NR1H3
IL2
CYSLTR2
CYBA
ABCC9
FGF4
AREG
SLC17A5
IRF9
AGGF1
SERPINA4
CD69
DDT
RNF182
HSPA12B
MIR141
TGFB1
ITGB2
LPL
MAPK9
SERPINC1
NFE2L2
PPARGC1A
SMAD2
PRDX5
CRK
LOX
APEX1
PTGDS
COX5A
CX3CL1
TRAP1
HMGB2
PPIG
CMKLR1
GRK2
BCL2L15
UCA1
SLC9A1
SLC24A3
HMGCR
