# Supplementary material for: Systematic Pharmacology Reveals the Antioxidative Stress and Anti-Inflammatory Mechanisms of Resveratrol Intervention in Myocardial Ischemia-Reperfusion Injury
Source: Evid Based Complement Alternat Med. 2021 May 21;2021:5515396. doi: 10.1155/2021/5515396 (PMC8163539; doi:10.1155/2021/5515396)
Supplement: Supplementary Materials — Table S1: predicted potential targets of resveratrol. Table S2: MIRI genes. Table S3: enrichment analysis of Resveratrol-MIRI PPI Network. Table S4: Reactome pathway of Resveratrol-MIRI PPI. [file 5515396.f1.zip › 5515396.f1/Table S3.pdf]

Table S3 Enrichment Analysis of Resveratrol-MIRI PPI Network

Category	Term	Description	Count	%	PValue	Genes
**Biological Proc**	GO:0043401	steroid horm	21	8.571429	4.98E-23	RARG, THRB,
	GO:0071222	cellular resp	22	8.979592	9.21E-18	IL10, NOS2, SR
	GO:0043066	negative regu	36	14.69388	4.96E-16	GSK3B, UCN,
	GO:0070374	positive regu	21	8.571429	9.07E-13	MAP2K1, TGF
	GO:0008284	positive regu	32	13.06122	1.21E-12	RARG, ADM,
	GO:0007568	aging	20	8.163265	3.00E-12	IL10, NQO1, T
	GO:0050900	leukocyte mi	17	6.938776	2.24E-11	SRC, ITGB2, F
	GO:0018108	peptidyl-tyro	18	7.346939	7.69E-11	MAP2K1, HSP
	GO:0030522	intracellular	11	4.489796	1.07E-10	AR, THRB, TH
	GO:0010628	positive regu	22	8.979592	2.19E-10	TGFB2, MAP2
	GO:0045429	positive regu	11	4.489796	4.06E-10	HSP90AA1, HS
	GO:0014068	positive regu	12	4.897959	2.00E-09	SELP, TGFB2,
	GO:0001666	response to h	17	6.938776	3.91E-09	TGFB2, TGFB1
	GO:0000187	activation of	14	5.714286	4.58E-09	MAP2K1, INSR
	GO:0045909	positive regu	9	3.673469	5.48E-09	UCN, GJA1, N
	GO:0006954	inflammator	24	9.795918	6.72E-09	IL10, UCN, TG
	GO:0002576	platelet degr	13	5.306122	2.94E-08	CFD, TGFB2, T
	GO:0051897	positive regu	12	4.897959	3.27E-08	TGFB1, F10, S
	GO:0032496	response to l	15	6.122449	1.11E-07	MAOB, ABCC
	GO:0007169	transmembra	12	4.897959	1.33E-07	ITK, ZAP70, S
	GO:0048384	retinoic acid	7	2.857143	1.35E-07	RXRB, RARG,
	GO:0032869	cellular resp	11	4.489796	1.51E-07	PDPK1, STAT1
	GO:0018105	peptidyl-seri	13	5.306122	2.56E-07	GSK3B, SYK,
	GO:0000165	MAPK casca	18	7.346939	2.65E-07	MAP2K1, TGF
	GO:0043627	response to e	10	4.081633	3.66E-07	DTYMK, HSP9
	GO:0051384	response to g	10	4.081633	3.66E-07	IL10, UCN, BC
	GO:0030335	positive regu	15	6.122449	4.60E-07	TGFB1, F10, IN
	GO:0010629	negative regu	13	5.306122	6.93E-07	SMAD2, UCN,
	GO:0030168	platelet activ	12	4.897959	8.45E-07	ADAMTS13, S
	GO:0071456	cellular resp	11	4.489796	1.22E-06	CCNA2, EPAS1
	GO:0007173	epidermal gr	9	3.673469	1.30E-06	ADAM17, TGF
	GO:0022617	extracellular	10	4.081633	1.42E-06	MMP12, MMP1
	GO:0043406	positive regu	9	3.673469	1.95E-06	TGFB1, SRC, K
	GO:0048661	positive regu	9	3.673469	2.22E-06	STAT1, AKR1
	GO:2000352	negative regu	7	2.857143	2.43E-06	IL10, ABL1, K
	GO:0008202	steroid meta	8	3.265306	2.45E-06	SULT2B1, CYP
	GO:0030593	neutrophil ch	9	3.673469	4.63E-06	TGFB2, SYK, I
	GO:0008285	negative regu	20	8.163265	4.97E-06	IL10, SMAD2,
	GO:0032930	positive regu	5	2.040816	5.01E-06	TGFB1, GSTP1
	GO:0001938	positive regu	9	3.673469	6.50E-06	BMP2, ARG1,
	GO:0043065	positive regu	17	6.938776	7.66E-06	RARG, TGFB1,
	GO:0048010	vascular end	9	3.673469	8.96E-06	HSP90AA1, SR
	GO:0050852	T cell recept	12	4.897959	1.00E-05	ITK, ZAP70, IF
	GO:0055114	oxidation-re	24	9.795918	1.62E-05	NQO1, MAOB,

GO:0006805	xenobiotic m	9	3.673469	1.63E-05	NQO1, CYP2C9
GO:0014066	regulation of	9	3.673469	1.63E-05	ERBB4, LCK, K
GO:0070301	cellular respo	8	3.265306	1.69E-05	PPP5C, ARG1,
GO:0050728	negative regu	9	3.673469	1.79E-05	NR1H4, NR1H3
GO:0000302	response to r	7	2.857143	1.84E-05	PRDX5, GSTP1
GO:0001525	angiogenesis	14	5.714286	2.19E-05	TGFB2, SYK, N
GO:0006809	nitric oxide b	5	2.040816	2.72E-05	NQO1, NOS2, N
GO:0060334	regulation of	5	2.040816	2.72E-05	HSP90AB1, IFN
GO:0071356	cellular respo	10	4.081633	3.03E-05	ADAMTS13, K
GO:0045766	positive regu	10	4.081633	4.31E-05	CYSLTR2, NOS
GO:0043388	positive regu	6	2.44898	4.47E-05	HMGB2, IGF1,
GO:0032868	response to i	8	3.265306	4.93E-05	IL10, FABP3, A
GO:0060397	JAK-STAT c	5	2.040816	5.07E-05	PTPN1, STAT3
GO:1904707	positive regu	5	2.040816	5.07E-05	IL10, MMP2, M
GO:0045087	innate immu	19	7.755102	5.42E-05	ITK, SYK, SRC
GO:0010887	negative regu	4	1.632653	5.73E-05	NR1H2, NR1H3
GO:0002740	negative regu	4	1.632653	5.73E-05	IL10, APOA2, A
GO:0045776	negative regu	6	2.44898	6.33E-05	UCN, NOS2, NO
GO:0050729	positive regu	8	3.265306	8.59E-05	PLA2G2A, LPL
GO:0042523	positive regu	5	2.040816	8.64E-05	ERBB4, KIT, IC
GO:0031663	lipopolysacc	6	2.44898	8.74E-05	TGFB1, NOS3,
GO:0033189	response to v	5	2.040816	1.10E-04	ARG1, CAT, RA
GO:0060324	face develop	5	2.040816	1.10E-04	MAP2K1, RAR
GO:0045454	cell redox ho	8	3.265306	1.21E-04	PRDX5, NOS2,
GO:0051092	positive regu	10	4.081633	1.33E-04	AR, TGFB1, IT
GO:0043410	positive regu	8	3.265306	1.66E-04	AR, BMP2, INS
GO:0006928	movement of	8	3.265306	2.42E-04	MAP2K1, IFNG
GO:1901215	negative regu	6	2.44898	2.60E-04	UCN, PPP5C, S
GO:0043124	negative regu	6	2.44898	2.60E-04	STAT1, GSTP1
GO:0007179	transforming	8	3.265306	3.66E-04	SMAD2, FKBP
GO:0002523	leukocyte mi	4	1.632653	4.48E-04	ITGB2, S100A9
GO:0098869	cellular oxid	7	2.857143	5.11E-04	PRDX5, GSTP1
GO:0042110	T cell activat	6	2.44898	5.59E-04	FKBP1A, DPP4
GO:0032760	positive regu	6	2.44898	5.59E-04	IFNG, CYBA, H
GO:0002224	toll-like rece	5	2.040816	5.69E-04	CTSK, MAPKA
GO:0019430	removal of su	4	1.632653	5.91E-04	NQO1, NOS3, N
GO:0007204	positive regu	9	3.673469	7.26E-04	GJA1, ABL1, A
GO:0043542	endothelial c	5	2.040816	7.53E-04	DPP4, STAT1, I
GO:0010745	negative regu	4	1.632653	7.60E-04	NR1H2, NR1H3
GO:2001275	positive regu	4	1.632653	7.60E-04	AKT2, NR1H4,
GO:0042542	response to h	6	2.44898	8.16E-04	STAT1, MB, SP
GO:2000379	positive regu	5	2.040816	8.59E-04	AKR1C3, F2, M
GO:0043552	positive regu	5	2.040816	9.75E-04	TGFB1, ERBB4
GO:0007597	blood coagul	4	1.632653	0.002055	F10, F11, F2, K
GO:0038128	ERBB2 signa	5	2.040816	0.002114	HSP90AA1, ER

**Cell Componer**	GO:0005829	cytosol	114	46.53061	1.77E-24	PNMT, EPRS, I
	GO:0005615	extracellular	68	27.7551	6.59E-22	UCN, SERPINA
	GO:0005576	extracellular	62	25.30612	3.56E-14	SERPINA1, AD
	GO:0070062	extracellular	82	33.46939	2.42E-12	SERPINA1, TH
	GO:0005654	nucleoplasm	76	31.02041	5.68E-10	RARG, THRB,
	GO:0009986	cell surface	27	11.02041	1.81E-08	HSP90AB1, ITC
	GO:0031234	extrinsic con	10	4.081633	2.86E-07	ITK, ZAP70, SY
	GO:0045121	membrane ra	15	6.122449	7.24E-07	TNF, EGFR, TC
	GO:0005739	mitochondric	41	16.73469	1.03E-06	GSK3B, DTYM
	GO:0005737	cytoplasm	103	42.04082	5.40E-06	EPRS, NR3C1,
	GO:0031093	platelet alpha	8	3.265306	8.09E-06	CFD, TGFB2, T
	GO:0000790	nuclear chror	13	5.306122	1.13E-05	SMAD2, RARG
	GO:0005886	plasma mem	85	34.69388	1.29E-05	GSK3B, ITGB2
	GO:0005925	focal adhesic	18	7.346939	2.10E-05	MAP2K1, MMI
	GO:0043235	receptor com	10	4.081633	5.20E-05	RXRA, ERBB4,
	GO:0005634	nucleus	101	41.22449	8.70E-05	RORA, NR3C1,
	GO:0048471	perinuclear r	22	8.979592	9.37E-05	GSK3B, HSP90
	GO:0072562	blood microp	10	4.081633	2.06E-04	BCHE, TGFB1,
	GO:0043025	neuronal cell	14	5.714286	3.29E-04	NQO1, GSK3B,
	GO:0043234	protein comp	16	6.530612	4.44E-04	HSP90AA1, SY
	GO:0016324	apical plasma	13	5.306122	5.58E-04	HSP90AA1, HS
	GO:0090575	RNA polyme	5	2.040816	9.31E-04	RXRA, VDR, S
	GO:0036021	endolysosom	3	1.22449	0.001044	CTSK, CTSS, C
	GO:0005788	endoplasmic	10	4.081633	0.001121	BACE1, BCHE,
	GO:0005901	caveola	6	2.44898	0.001743	SRC, NOS3, IN
**Molecular Fun**	GO:0003707	steroid horm	24	9.795918	3.41E-28	RARG, THRB,
	GO:0004879	RNA polyme	15	6.122449	3.35E-17	NR1H2, STAT3
	GO:0004713	protein tyros	21	8.571429	4.66E-15	MAP2K1, HSP
	GO:0005515	protein bindi	183	74.69388	2.86E-13	RORA, EPRS, N
	GO:0005102	receptor binc	28	11.42857	1.62E-12	ITK, SRC, LPL,
	GO:0008144	drug binding	15	6.122449	3.71E-12	HSP90AB1, GS
	GO:0019899	enzyme bind	25	10.20408	1.04E-10	THRB, SRC, EC
	GO:0005524	ATP binding	55	22.44898	2.02E-10	GSK3B, ITK, H
	GO:0008270	zinc ion bind	46	18.77551	1.26E-09	RARG, THRB,
	GO:0004672	protein kinas	24	9.795918	2.56E-09	GSK3B, MAP2I
	GO:0005496	steroid bindi	9	3.673469	2.98E-09	AR, SULT1E1,
	GO:0008134	transcription	20	8.163265	3.32E-08	SMAD2, THRA
	GO:0043565	sequence-spe	27	11.02041	3.60E-08	RARG, THRB,
	GO:0004716	receptor sign	6	2.44898	1.44E-07	SYK, ERBB4, I
	GO:0003708	retinoic acid	5	2.040816	2.11E-07	RARG, RXRA,
	GO:0004715	non-membra	9	3.673469	2.76E-07	ITK, ZAP70, SY
	GO:0019903	protein phos	10	4.081633	2.85E-07	HSP90AA1, LC
	GO:0004714	transmembra	8	3.265306	1.04E-06	ERBB4, INSR, I
	GO:0046965	retinoid X re	6	2.44898	1.62E-06	RARG, VDR, N
	GO:0019901	protein kinas	20	8.163265	2.46E-06	PTPN1, TRAP1

	GO:0032052	bile acid bin	5	2.040816	2.85E-06	FABP6, AKR1
	GO:0046934	phosphatidyl	9	3.673469	2.93E-06	ERBB4, LCK,
	GO:0004252	serine-type e	16	6.530612	4.75E-06	CFD, F10, MM
	GO:0044325	ion channel	11	4.489796	5.59E-06	FKBP1A, DIAP
**Signaling Path**	hsa04931	Insulin resist	23	9.387755	3.02E-13	PTPN1, GSK3B
	hsa04068	FoxO signali	24	9.795918	3.79E-12	IL10, SMAD2,
	hsa04920	Adipocytoki	15	6.122449	9.63E-09	STAT3, PTPN1
	hsa04910	Insulin signa	20	8.163265	1.45E-08	PTPN1, GSK3B
	hsa04151	PI3K-Akt si	32	13.06122	1.51E-08	GSK3B, HSP90
	hsa04012	ErbB signali	16	6.530612	2.37E-08	GSK3B, MAP2
	hsa04660	T cell recept	17	6.938776	2.41E-08	IL10, GSK3B, I
	hsa03320	PPAR signal	14	5.714286	4.86E-08	PDPK1, APOA
	hsa04014	Ras signalin	24	9.795918	1.4E-07	MAP2K1, INSR
	hsa04915	Estrogen sig	16	6.530612	1.42E-07	KCNJ5, MAP2
	hsa04668	TNF signalir	16	6.530612	4.05E-07	MAP2K1, MM
	hsa04066	HIF-1 signal	15	6.122449	6.1E-07	MAP2K1, NOS
	hsa04015	Rap1 signali	21	8.571429	2.76E-06	MAP2K1, SRC,
	hsa04620	Toll-like rec	14	5.714286	1.12E-05	MAP2K1, STA
	hsa04062	Chemokine s	18	7.346939	2.73E-05	GSK3B, ITK, M
	hsa04520	Adherens jur	11	4.489796	3.54E-05	SMAD2, PTPN
	hsa04010	MAPK signa	21	8.571429	4.41E-05	TGFB2, MAP2
	hsa04370	VEGF signal	10	4.081633	6.03E-05	MAP2K1, SRC,
	hsa04960	Aldosterone-	8	3.265306	0.000114	SLC9A3R2, PD
	hsa04630	Jak-STAT si	14	5.714286	0.000299	IL10, STAT1, S
	hsa04670	Leukocyte tr	12	4.897959	0.000503	ITK, MMP2, IT
	hsa04024	cAMP signal	16	6.530612	0.000644	MAP2K1, PDE
	hsa04150	mTOR signa	8	3.265306	0.001392	PDPK1, AKT2,
	hsa04152	AMPK signa	11	4.489796	0.003084	CCNA2, PDPK
	hsa04064	NF-kappa B	9	3.673469	0.003741	ZAP70, SYK, L
	hsa00590	Arachidonic	7	2.857143	0.00843	CYP2C9, CYP2
	hsa04350	TGF-beta si	8	3.265306	0.0111	SMAD2, TGFB
	hsa04611	Platelet activ	10	4.081633	0.013592	PPP1CC, SYK,
	hsa04610	Complement	7	2.857143	0.01503	CFD, SERPINA
	hsa04390	Hippo signal	10	4.081633	0.0324	SMAD2, GSK3

old Enrichmen	FDR
25.45895603	1.22E-19
13.45365818	5.66E-15
5.467480667	2.43E-13
8.292345679	3.71E-10
4.745262191	4.24E-10
8.376106746	8.59E-10
9.629089928	4.57E-09
8.129750666	1.45E-08
20.00346545	1.87E-08
5.802531964	3.58E-08
17.6774811	5.86E-08
12.75745489	2.46E-07
6.829935879	4.37E-07
9.041498404	4.89E-07
21.44572158	5.61E-07
4.37590801	6.60E-07
8.721722802	2.77E-06
9.871840094	2.97E-06
6.320385426	9.76E-06
8.637860082	1.11E-05
26.87334248	1.11E-05
9.871840094	1.20E-05
7.186699588	1.93E-05
4.747526152	1.93E-05
10.63121241	2.37E-05
10.63121241	2.37E-05
5.63338701	2.90E-05
6.557207654	4.26E-05
7.210735373	5.06E-05
7.918038409	6.99E-05
11.10582011	7.24E-05
9.092484297	7.76E-05
10.54111739	1.04E-04
10.3654321	1.16E-04
17.27572016	1.23E-04
12.85634989	1.23E-04
9.42312009	2.11E-04
3.490044478	2.20E-04
38.39048925	2.20E-04
9.013419216	2.62E-04
3.915829904	3.03E-04
8.637860082	3.44E-04
5.60293627	3.78E-04
2.801468135	5.48E-04

7.973409307	5.48E-04
7.973409307	5.48E-04
9.698649917	5.62E-04
7.872480075	5.87E-04
12.40308114	5.96E-04
4.33829744	6.99E-04
26.57803102	8.24E-04
26.57803102	8.24E-04
6.28208006	9.08E-04
6.008946144	0.00127628
14.80776014	0.00130723
8.251090228	0.00142537
23.03429355	0.00143106
23.03429355	0.00143106
3.053383099	0.00151316
46.06858711	0.00154545
46.06858711	0.00154545
13.82057613	0.00164143
7.572918428	0.00216539
20.32437666	0.00216539
12.95679012	0.00216835
19.19524463	0.00267092
19.19524463	0.00267092
7.179520068	0.00290742
5.195705313	0.00318125
6.824975867	0.00374687
6.428174945	0.00503247
10.3654321	0.00532195
10.3654321	0.00532195
6.008946144	0.00710253
25.12832024	0.00826931
6.910288066	0.00890273
8.821644339	0.0095568
8.821644339	0.0095568
12.79682975	0.0095568
23.03429355	0.0096083
4.641238253	0.0115855
11.91428977	0.01181073
21.26242482	0.01181073
21.26242482	0.01181073
8.129750666	0.01253015
11.51714678	0.01310191
11.14562591	0.01442628
15.3561957	0.02699601
9.092484297	0.02745582

2.568474149	5.05E-22
3.770467463	9.39E-20
2.876204053	3.38E-12
2.178747427	1.72E-10
2.038910872	3.24E-08
3.720646059	8.58E-07
10.98360656	1.16E-05
5.4384848	2.58E-05
2.300698353	3.27E-05
1.473174652	1.54E-04
10.86378539	2.10E-04
5.030833263	2.68E-04
1.540530112	2.82E-04
3.438346401	4.28E-04
5.880986188	9.87E-04
1.393082361	0.00155009
2.645970275	0.00157136
4.913718723	0.00326509
3.319489982	0.00494065
2.900525227	0.00632543
3.336600755	0.00757659
11.31644312	0.01206342
56.01639344	0.01293415
3.890027322	0.01331694
6.894325347	0.01986632
29.52944606	2.04E-25
28.70918367	1.00E-14
10.8792696	9.31E-13
1.435295785	4.28E-11
5.465317685	1.94E-10
13.599087	3.70E-10
5.172825887	8.86E-09
2.534857689	1.51E-08
2.711286465	8.36E-08
4.606264567	1.53E-07
22.96734694	1.62E-07
4.852256396	1.66E-06
3.591419116	1.66E-06
41.34122449	6.16E-06
68.90204082	8.41E-06
13.48083407	1.00E-05
10.93683188	1.00E-05
14.5056928	3.47E-05
27.56081633	5.09E-05
3.665002171	7.36E-05

43.06377551	7.98E-05
10.00190915	7.98E-05
4.323265306	1.24E-04
6.70727831	1.39E-04
7.252337734	2.09E-11
6.099305453	1.04E-10
7.29738331	9.49E-08
4.935428325	1.23E-07
3.158674128	1.23E-07
6.262888358	1.66E-07
5.789257426	1.66E-07
7.115856362	3.05E-07
3.616402348	7.84E-07
5.503750375	7.84E-07
5.09225502	1.99E-06
5.321008663	2.59E-06
3.405445545	9.76E-06
4.497758266	3.4309E-05
3.295592462	7.85E-05
5.276042393	9.39E-05
2.826654405	1.15E-04
5.582697614	1.49E-04
6.985529322	2.71E-04
3.288016388	6.15E-04
3.553508394	0.00100581
2.751875188	0.00123348
4.697166268	0.00249437
3.045520406	0.00545561
3.522874701	0.00653458
3.90788833	0.01384901
3.243281471	0.01721121
2.619573496	0.02061159
3.454799828	0.02138244
2.25526195	0.04299172
