# Supplementary material for: Systematic Pharmacology Reveals the Antioxidative Stress and Anti-Inflammatory Mechanisms of Resveratrol Intervention in Myocardial Ischemia-Reperfusion Injury
Source: Evid Based Complement Alternat Med. 2021 May 21;2021:5515396. doi: 10.1155/2021/5515396 (PMC8163539; doi:10.1155/2021/5515396)
Supplement: Supplementary Materials — Table S1: predicted potential targets of resveratrol. Table S2: MIRI genes. Table S3: enrichment analysis of Resveratrol-MIRI PPI Network. Table S4: Reactome pathway of Resveratrol-MIRI PPI. [file 5515396.f1.zip › 5515396.f1/Table S4.pdf]

Table S4 Reactome Pathway of Resveratrol-MIRI PPI Network

Description	Observed ge	Backgrou	Strength	Genes and	False discovery rate
Signal Transdu	97	2605	0.47	CX3CL1,P	3.49E-21
Nuclear Recep	19	39	1.59	NR1H2,RA	8.83E-20
Immune Syste	79	1925	0.52	MIF,MAPI	4.24E-19
Signaling by I	39	439	0.85	MIF,MAPI	1.46E-18
Metabolism	79	2032	0.49	PYGL,STS	6.25E-18
Hemostasis	43	601	0.76	MIF,MAPI	1.74E-17
Innate Immune	54	1012	0.63	MIF,MAPI	4.83E-17
Cytokine Sign	42	654	0.71	MIF,MAPI	1.48E-15
Signaling by F	35	437	0.81	FGF4,MAI	1.64E-15
Diseases of sig	31	360	0.84	FGF4,MAI	1.87E-14
SUMOylation	13	23	1.65	NR3C1,NF	1.92E-14
Generic Trans	52	1112	0.57	MAPK1,A	2.95E-14
Metabolism of	41	721	0.66	STS,SULT	1.25E-13
Platelet activa	25	256	0.89	MAPK1,T	9.46E-13
Interleukin-4 a	18	106	1.13	MMP2,TG	1.07E-12
Gene expressi	53	1366	0.49	MAPK1,A	1.54E-11
PI3K/AKT Sig	14	85	1.12	FGF4,MDI	1.15E-09
Toll-like Rece	17	151	0.95	MAPK1,M	1.79E-09
SUMO E3 liga	17	154	0.95	AURKA,N	2.26E-09
Cellular respo	25	384	0.72	MAPK1,M	2.66E-09
Disease	41	1018	0.51	FGF4,MAI	3.22E-09
Neutrophil deg	27	471	0.66	MIF,MAPI	5.71E-09
Signaling by N	17	167	0.91	PPP5C,MA	5.79E-09
Negative regul	13	92	1.05	FGF4,MAI	2.31E-08
Signaling by F	10	43	1.27	RARA,PPA	3.23E-08
Signaling by V	13	104	1	MAPK14,C	8.05E-08
PI5P, PP2A an	12	85	1.05	FGF4,MAI	9.39E-08
Extracellular m	20	298	0.73	MMP2,TG	0.000000101
PIP3 activates	18	242	0.77	FGF4,MAI	0.000000139
Intracellular si	19	274	0.74	FGF4,MAI	0.000000145
Signaling by S	9	40	1.25	STAT3,KI	0.000000235
VEGFA-VEG	12	95	1	MAPK14,C	0.000000251
TRIF(TICAM	12	96	1	MAPK1,M	0.000000271
Constitutive S	10	58	1.14	FGF4,EGF	0.000000291
Metabolism of	14	146	0.88	AKR1B1,A	0.000000329
Interleukin-2 f	9	43	1.22	IL2,STAT3	0.000000346
Toll Like Rece	13	126	0.92	MAPK1,M	0.000000471
